# Overexpressed methyltransferase-like 1 (METTL1) increased chemosensitivity of colon cancer cells to cisplatin by regulating miR-149-3p/S100A4/p53 axis

**DOI:** 10.18632/aging.102575

**Published:** 2019-12-20

**Authors:** Yang Liu, Chunyan Yang, Yong Zhao, Qiang Chi, Zhen Wang, Boshi Sun

**Affiliations:** 1 The 3rd Department of General Surgery, The 2nd Affiliated Hospital of Harbin Medical University, Harbin 150001, Heilong Jiang, China; 2 Department of Oral and Maxillofacial Surgery, The 2nd Affiliated Hospital of Harbin Medical University, Harbin 150001, Heilong Jiang, China

**Keywords:** colon cancer, METTL1, cisplatin, miR-149-3p, S100A4

## Abstract

Methyltransferase-like 1 (METTL1) mediated 7-methylguanosine (m7G) is crucial for the regulation of chemoresistance in cancer treatment. However, the role of METTL1 in regulating chemoresistance of colon cancer (CC) cells to cisplatin is still unclear. This study established the cisplatin-resistant CC (CR-CC) cells and found that METTL1 was low-expressed in CR-CC cells compared to their paired cisplatin-sensitive CC (CS-CC) cells. Besides, overexpressed METTL1 enhanced the cytotoxic effects of cisplatin on CR-CC cells. In addition, miR-149-3p was the downstream target of METTL1, which could be positively regulated by METTL1. Further results validated that miR-149-3p was low-expressed in CR-CC cells comparing to the CS-CC cells. In addition, the promoting effects of overexpressed METTL1 on cisplatin induced CR-CC cell death were abrogated by synergistically knocking down miR-149-3p. Furthermore, S100A4/p53 axis was the downstream target of METTL1 and miR-149-3p, and either overexpressed METTL1 or miR-149-3p increased p53 protein levels in CR-CC cells, which were reversed by upregulating S100A4. Similarly, the promoting effects of overexpressed METTL1 on cisplatin-induced CR-CC cell death were abrogated by overexpressing S100A4. Taken together, overexpression of METTL1 sensitized CR-CC cells to cisplatin by modulating miR-149-3p/S100A4/p53 axis.

## INTRODUCTION

Cisplatin was the first-line chemotherapeutic drug for colon cancer (CC) treatment in clinic [1, 2]. However, continuous cisplatin treatment induced chemoresistance of CC cells to cisplatin, which seriously limited its therapeutic efficacy [3–5], but the underlying mechanisms were still not fully delineated. Methyltransferase-like 1 (METTL1) was crucial for RNA processing (splicing, stability and localization) in a 7-methylguanosine (m7G) dependent manner [6–8], which served as a tumor suppressor and participated in the development of multiple cancers [9, 10]. Notably, METTL1 also determined sensitivity of cervical cancer cells to 5-fluorouracil (5-FU) [9], however, it is still unclear whether METTL1 participated in the regulation of chemoresistance of CC cells to Cisplatin.

MicroRNAs (miRNAs) were closely related with cancer progression and regulated chemoresistance generated by cancer cells to cisplatin according to their biological functions [11–13]. Recent studies have identified miR-149-3p as a tumor suppressor in multiple cancers, such as prostate cancer [14, 15], pancreatic cancer [16], colon cancer [17] and so on. Besides, overexpressed miR-149-3p abrogated cisplatin-resistance of ovarian cancer cells [18], esophageal cancer cells [19], gastric cancer cells [20] and non-small cell lung cancer cells [21], however, only a few publications reported the role of miR-149-3p in regulating CC cells’ resistance to cisplatin. In addition, previous studies proved that microRNAs could be regulated by METTL1 in a m7G dependent manner [22], and miR-149-3p was the potential downstream target of METTL1 [10], but the role of METTL1/miR-149-3p axis in regulating CC cell functions are still unclear.

S100A4 was a small calcium-binding protein and played an oncogenic role in the development of multiple cancers, such as prostate cancer [23], breast cancer [24] and colon cancer [25, 26]. Besides, S100A4 participated in the regulation of Cisplatin-resistance of different cancer cells in a p53-dependent manner [27, 28]. Since Cisplatin promoted cancer cell death and inhibited cell viability by inducing DNA double-strand break (DSB) [29, 30], and p53 was the hub gene of DSB-induced cell death [31], it was reasonable to hypothesize that S100A4/p53 axis was crucial for regulating Cisplatin-resistance of CC cells. Interestingly, previous study found that miR-149-3p inhibited bladder cancer cell proliferation and migration by downregulating S100A4 expression levels [15], which indicated that S100A4/p53 axis might be regulated by miR-149-3p.

Taken together, this study aimed to investigate the role of METTL1/miR-149-3p/S100A4/p53 cascade in the regulation of cisplatin-resistance of CC cells, which will provide new potential therapeutic agents for CC treatment in clinic.

## RESULTS

### The expression levels of METTL1 and miR-149-3p in CS-CC and CR-CC cells

**Figure 1:**
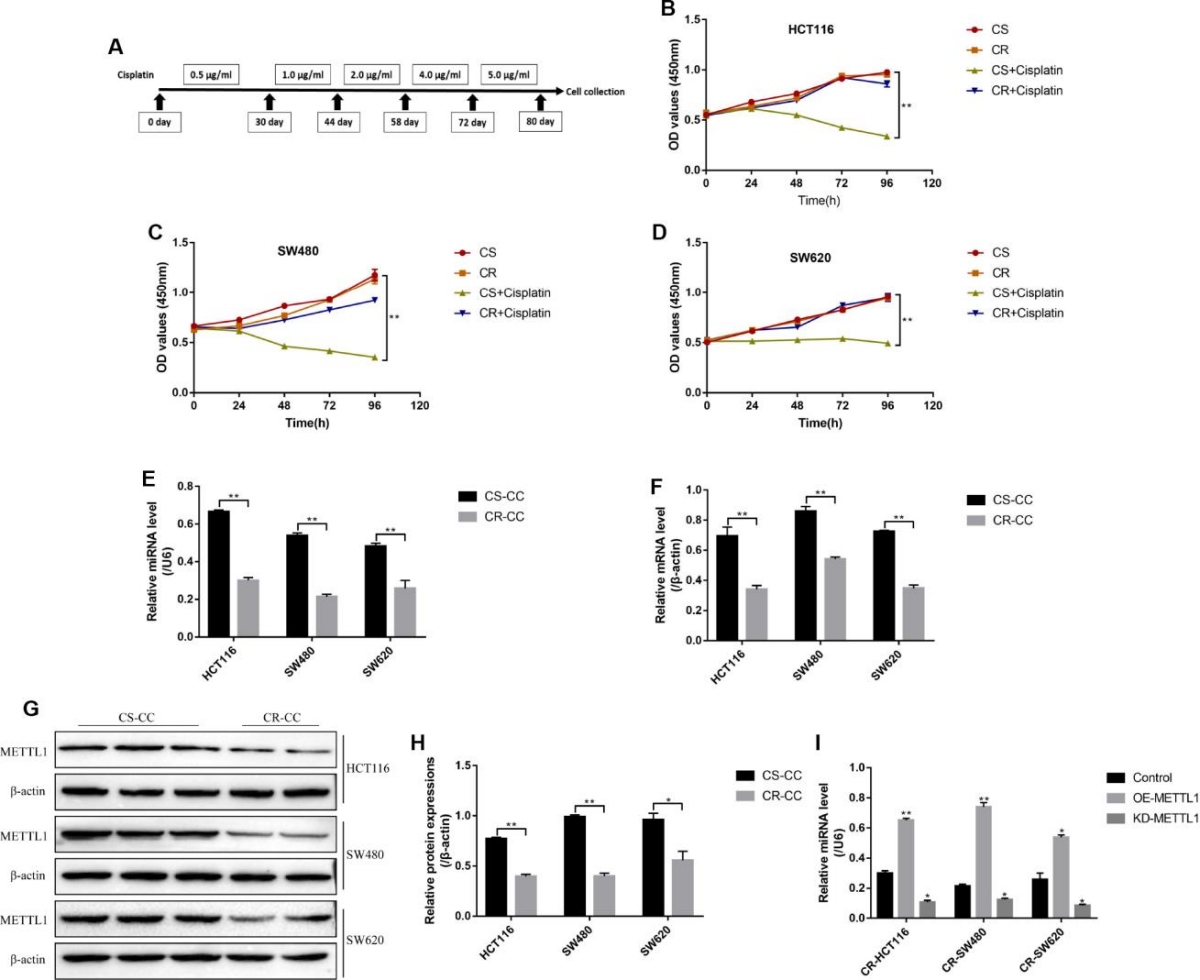
**The expression levels of METTL1 and miR-149-3p in CR-CC cell lines.** (**A**) The schematic procedures of the induction of CR-HCT116, CR-SW480 and CR-SW620 respectively. CCK-8 assay was conducted to detect (**B**) HCT116 cells, (**C**) SW480 cells and (**D**) SW620 cells proliferation respectively. Real-Time qPCR was performed to determine the levels of (**E**) miR-149-3p and (**F**) METTL1 mRNA in CC cells. (**G**, **H**) Western Blot was used to determine the expression levels of METTL1 in Cisplatin-sensitive colon cancer (CS-CC) and Cisplatin-resistant colon cancer (CR-CC) cells. (**I**) Real-Time qPCR was employed to investigate the effects of METTL1 on miR-149-3p levels in CR-CC cells. All the experiments repeated at least 3 times. “*” means *p* < 0.05, “**” means *p* < 0.01 and “NS” means no statistical significance.

The CC cells (HCT116, SW480 and SW620) were continuously exposed to low-dose cisplatin starting from 0.5μg/ml to 5μg/ml in a stepwise manner to induct the cisplatin-resistant CC (CR-CC) cell lines (Figure 1A). The CCK-8 results showed that the CR-CC cells were successfully inducted by this method (Figure 1B–1D). Specifically, high-dose cisplatin (20μg/ml) significantly inhibited CS-CC cell viability, but had little effects on CR-CC cell proliferation (Figure 1B–1D). Further results showed that both METTL1 mRNA (Figure 1F) and miR-149-3p (Figure 1E) were lowly expressed in CR-CC cells comparing to their paried CS-CC cells, and the Western Blot results validated that the expression levels of METTL1 were lower in CR-CC cells comparing to the CS-CC cells (Figure 1G–1H). In addition, METTL1 was successfully overexpressed and downregulated in CR-HCT116, CR-SW480 and CR-SW620 cells respectively (Supplementary Figure 1A–1B). The results showed that overexpressed METTL1 significantly increased miR-149-3p levels in CR-CC cells, and downregulation of METTL1 had opposite effects on miR-149-3p levels (Figure 1I). However, miR-149-3p did not regulate METTL1 expressions in CS-CC cells (Supplementary Figure1C, 1D), which were in accordance with the previous study [10] and indicated that METTL1 positively regulated miR-149-3p levels in CR-CC cells.

### Overexpressed METTL1 enhanced the cytotoxic effects of high-dose cisplatin on CR-CC cells by targeting miR-149-3p

**Figure 2:**
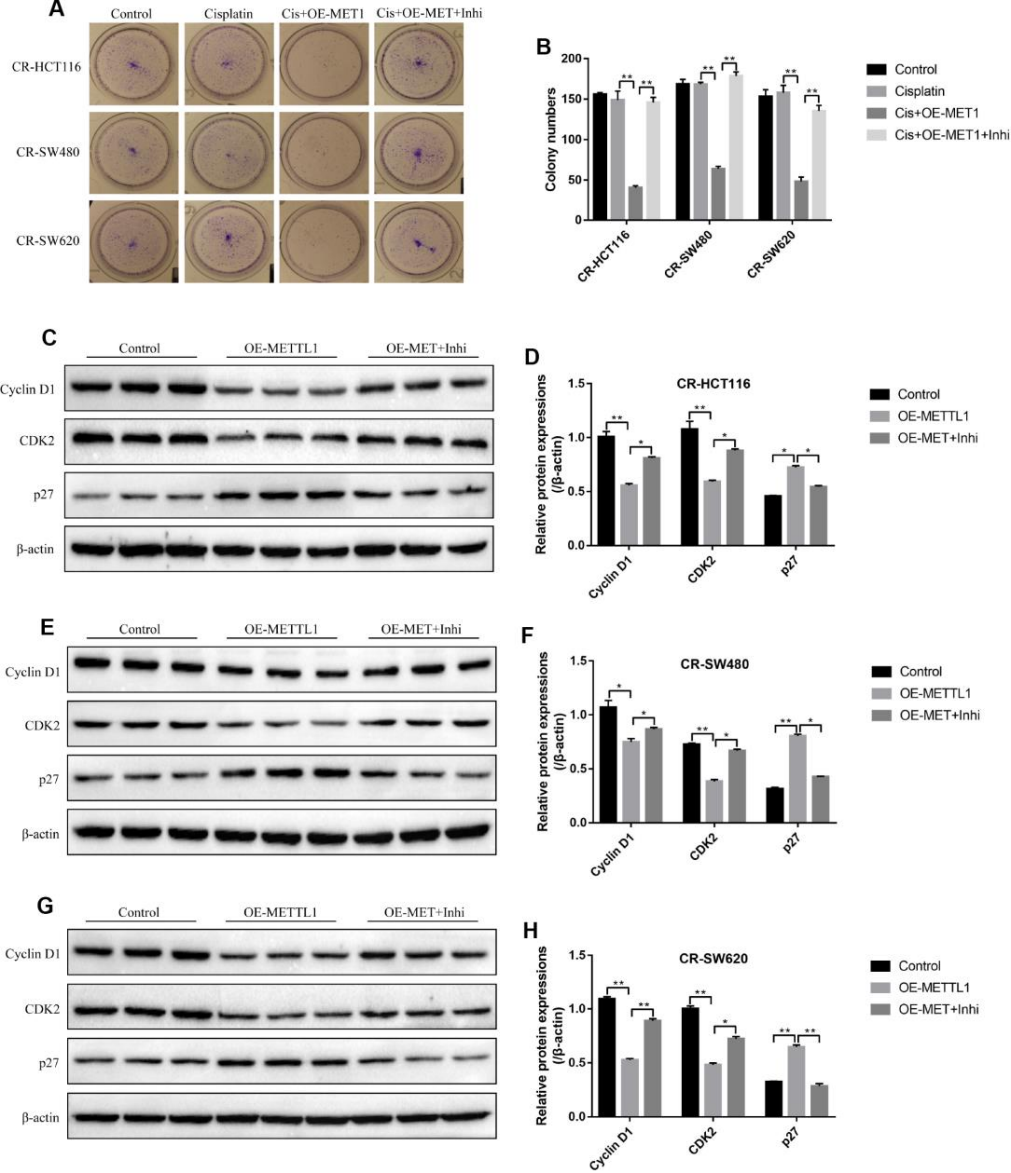
**METTL1 regulated CR-CC cell proliferation by targeting miR-149-3p.** (**A**, **B**) Colony formation assay was performed to evaluate the colony formation abilities in CC cells. Western Blot was conducted to detect the expression levels of Cyclin D1, CDK2 and p27 in (**C**, **D**) CR-HCT116 cells, (**E**, **F**) CR-SW480 cells and (**G**, **H**) CR-SW620 cells. (“OE-METTL1” represented “Overexpressed METTL1 group”, and “OE-METTL1+Inhi” represented “Overexpressed METTL1 plus miR-149-3p inhibitor group”). All the experiments repeated at least 3 times. “*” means *p* < 0.05, “**” means *p* < 0.01 and “NS” means no statistical significance.

**Figure 3:**
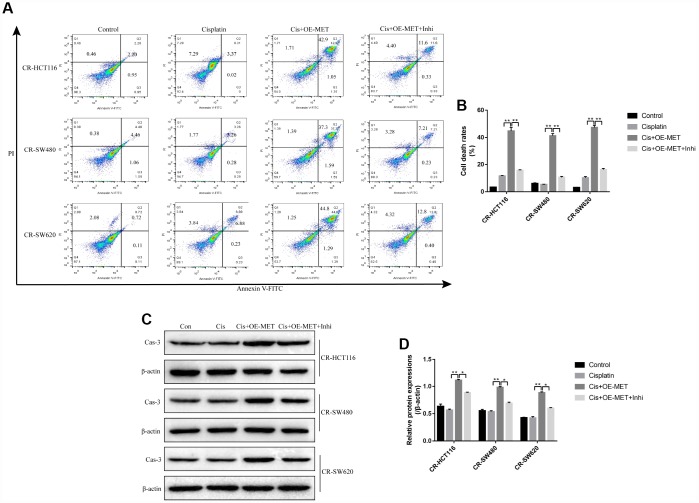
**The involvement of METTL1/miR-149-3p axis in the regulation of CR-CC cell apoptosis.** (**A**, **B**) FCM was employed to determine the apoptosis ratio of CR-CC cells. (**C**, **D**) Western Blot was used to detect the expression levels of cleaved Caspase-3 in CR-HCT116 cells, CR-SW480 cells and CR-SW620 cells respectively. (“Con” represented “Control group”, “Cis” indicated “Cisplatin treated group”, “Cis+OE-MET” represented “Cisplatin plus overexpressed METTL1 treated group”, “Cis+OE-MET+Inhi” represented “Cisplatin plus overexpressed METTL1 and miR-149-3p inhibitor treated group”). All the experiments repeated at least 3 times. “*” means *p* < 0.05, “**” means *p* < 0.01 and “NS” means no statistical significance.

Further experiments delved the effects of METTL1 on high-dose cisplatin induced CR-CC cell death. The colony formation assay results showed that overexpressed METTL1 significantly enhanced the inhibiting effects of high-dose cisplatin on CR-CC cell proliferation, which were abrogated by synergistically knocking down miR-149-3p (Figure 2A, 2B). In addition, upregulation of METTL1 decreased the expression levels of Cyclin D1 and CDK2, increased p27 expression levels in CR-HCT116 cells (Figure 2C, 2D), CR-SW480 cells (Figure 2E, 2F) and CR-SW620 cells (Figure 2G, 2H) respectively. Of note, the effects of overexpressed METTL1 on the above proteins were reversed by synergistically downregulating miR-149-3p (Figure 2C–2H). Further results showed that high-dose cisplatin (20 μg/ml) had little effects on CR-CC cell apoptosis ratio, which were significantly increased by synergistically overexpressing METTL1 in CR-CC cells (Figure 3A, 3B), and the effects of overexpressed METTL1 on cell apoptosis were abrogated by co-transfecting cells with miR-149-3p inhibitor (Figure 3A, 3B). In parallel, overexpressed METTL1 increased the expression levels of cleaved Caspase 3 in cisplatin treated CR-CC cells, which were also abrogated by knocking down miR-149-3p (Figure 3C, 3D).

### S100A4/p53 axis was regulated by METTL1 and miR-149-3p in CS-CC cells

**Figure 4:**
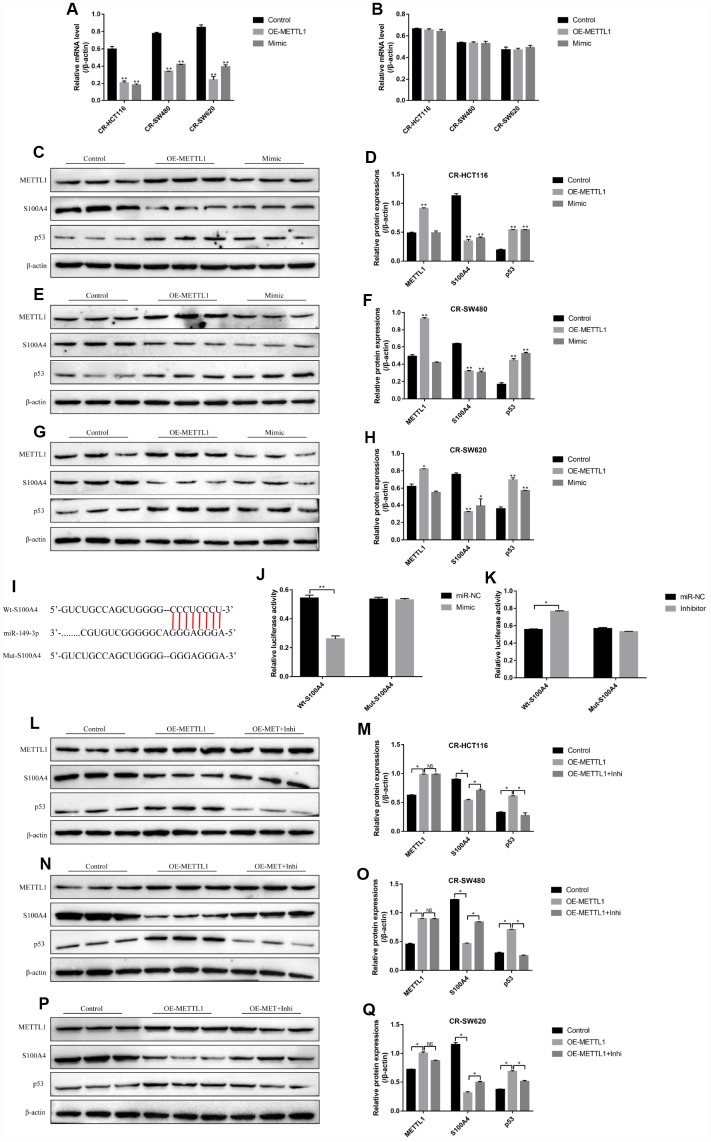
**S100A4/p53 axis was the downstream target of METTL1 and miR-149-3p.** Real-Time qPCR was conducted to detect the mRNA levels of (**A**) S100A4 and (**B**) p53 in CR-CC cells. Western Blot was performed to determine the expression levels of METTL1, S100A4 and p53 in (**C**, **D**) CR-HCT116 cells, (**E**, **F**) CR-SW480 cells and (**G**, **H**) CR-SW620 cells. (**I**) The binding sites of miR-149-3p and the 3′UTR regions of S100A4 were predicted by online starBase software. Western Blot was used to detect the expression levels of METTL1, S100A4 and p53 in (**L**, **M**) CR-HCT116 cells, (**N**, **O**) CR-SW480 cells and (**P**, **Q**) CR-SW620 cells. (“OE-METTL1” represented “Overexpressed METTL1 group”, and “OE-METTL1+Inhi” represented “Overexpressed METTL1 plus miR-149-3p inhibitor group”). All the experiments repeated at least 3 times. “*” means *p* < 0.05, “**” means *p* < 0.01 and “NS” means no statistical significance.

The results showed that either overexpressed METTL1 or miR-149-3p decreased S100A4 expression levels in both mRNA levels (Figure 4A) and protein levels (Figure 4C–4H), and merely increased p53 protein expression levels in CS-CC cells (Figure 4C–4H), but had little effects on p53 mRNA levels (Figure 4B). Besides, the online starBase software predicted the binding sites of miR-149-3p and 3’UTR regions of S100A4 (Figure 4I), and the dual-luciferase reporter gene syetem assay validated that miR-149-3p inhibited S100A4 expression levels by binding to its 3’ UTR regions (Figure 4J, 4K). In addition, knock-down of miR-149-3p abrogated the effects of overexpressed METTL1 on the expression levels of S100A4 and p53 in CR-HCT116 cells (Figure 4L–4M), CR-SW480 cells (Figure 4N–4O) and CR-SW620 cells (Figure 4P–4Q). Interestingly, the results showed that S100A4 was high-expressed, and p53 was low-expressed in CR-CC cells comparing to their paired CS-CC cells (Supplementary Figure 2).

### Targeting S100A4/p53 axis enhanced high-dose cisplatin induced CR-CC cell death

**Figure 5:**
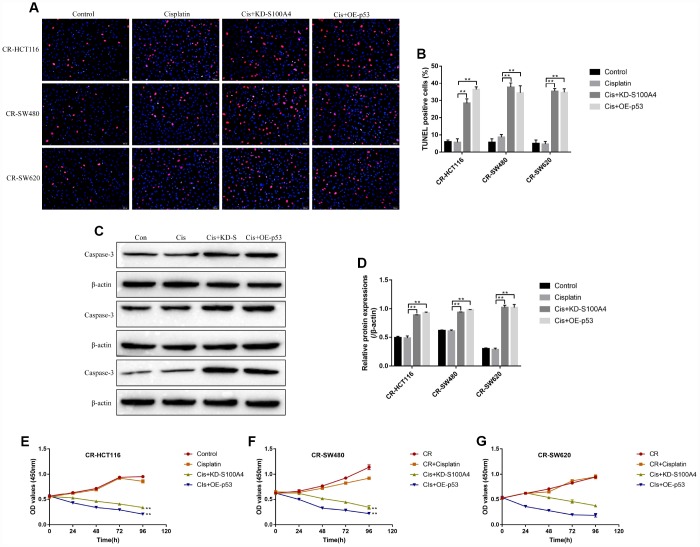
**The effects of S100A4 and p53 on CR-CC cell viability.** (**A**, **B**) TUNEL assay was conducted to detect cell apoptosis ratio in CC cells. (**C**, **D**) Western Blot was used to determine the expression levels of cleaved Caspase-3 in CR-CC cells. CCK-8 assay was performed to detect the proliferation ability of (**E**) CR-HCT116 cells, (**F**) CR-SW480 cells and (**G**) CR-SW620 cells. (“Con” represented “Control group”, “Cis” indicated “Cisplatin treated group”, “Cis+KD-S” represented “Cisplatin plus downregulated S100A4 treated group”, “Cis+OE-p53” represented “Cisplatin plus overexpressed p53 treated group”). All the experiments repeated at least 3 times. “*” means *p* < 0.05, “**” means *p* < 0.01 and “NS” means no statistical significance.

The study next explored the effects of S100A4/p53 axis on high-dose cisplatin induced CR-CC cell death. The results showed that upregulation of S100A4 decreased p53 protein levels (Supplementary Figure 3B–3G) instead of mRNA levels (Supplementary Figure 3A) in CS-CC cells, which was in line with the previous study and indirectly reflected that S100A4 promoted p53 protein degradation [28]. In addition, either downregulated S100A4 or upregulated p53 enhanced the cytotoxic effects of high-dose cisplatin on CR-CC cells (Figure 5). Specifically, the TUNEL assay results showed that the apoptosis ratio of cisplatin treated CR-CC cells was significantly increased by either knocking down S100A4 or overexpressing p53 (Figure 5A, 5B). Besides, the expression levels of cleaved Caspase 3 were increased by knocking down S100A4 or upregulating p53 in cisplatin treated CR-CC cells (Figure 5C, 5D). Furthermore, the CCK-8 results showed that either downregulated S100A4 or upregulated p53 enhanced the inhibiting effects of high-dose cisplatin treatment on CR-CC cells proliferation (Figure 5E–5G).

### The effects of METTL1 on cisplatin-induced CR-CC cell death were abrogated by upregulating S100A4

**Figure 6:**
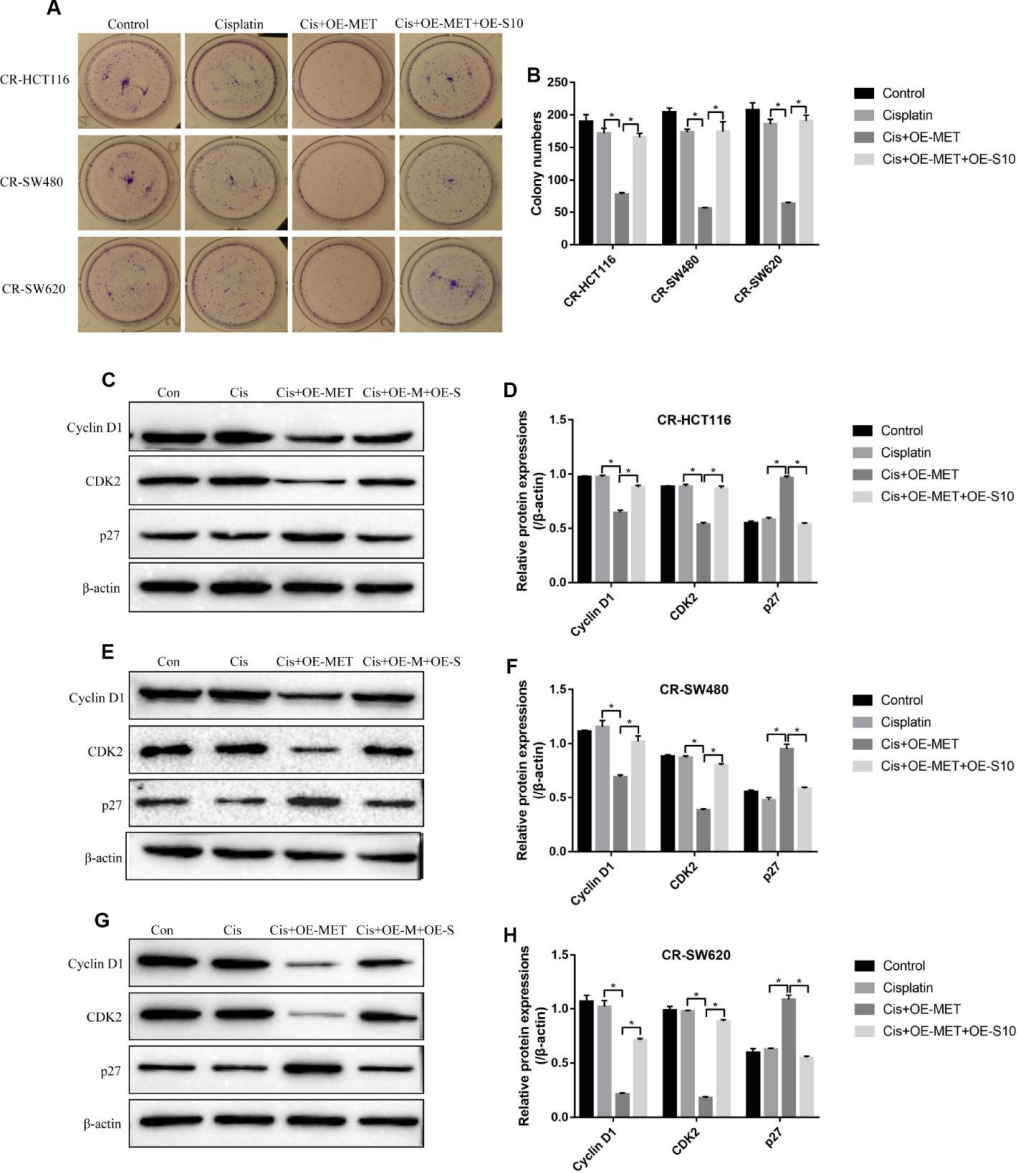
**METTL1 regulated cell proliferation by targeting S100A4.** (**A**, **B**) Colony formation assay was performed to detect cell proliferation. Western Blot was used to determine the expression levels of Cyclin D1, CDK2 and p27 in (**C**, **D**) CR-HCT116 cells, (**E**, **F**) CR-SW480 cells and (**G**–**H**) CR-SW620 cells. (“Con” represented “Control group”, “Cis” indicated “Cisplatin treated group”, “Cis+OE-MET” represented “Cisplatin plus overexpressed METTL1 treated group”, “Cis+OE-M+OE-S” represented “Cisplatin plus overexpressed METTL1 and overexpressed S100A4 treated group”). All the experiments repeated at least 3 times. “*” means *p* < 0.05, “**” means *p* < 0.01 and “NS” means no statistical significance.

**Figure 7:**
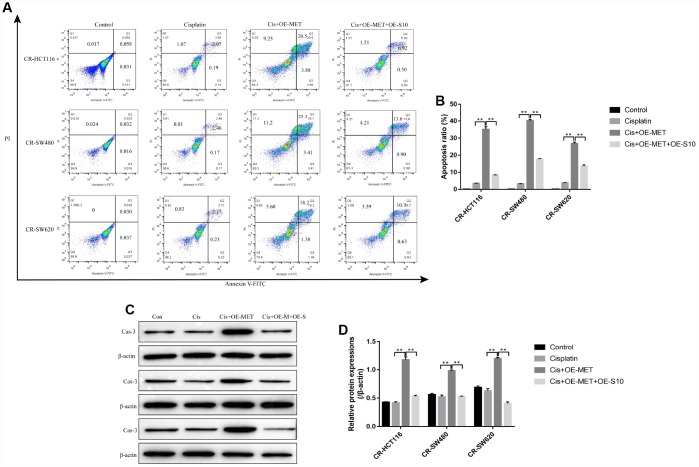
**Overexpressed METTL1 promoted CR-CC cells apoptosis by regulating S100A4.** (**A**, **B**) FCM was conducted to determine cell apoptosis ratio in CC cells. (**C**, **D**) Western Blot was employed to detect the expression levels of cleaved Caspase-3 in CR-CC cells. (“Con” represented “Control group”, “Cis” indicated “Cisplatin treated group”, “Cis+OE-MET” represented “Cisplatin plus overexpressed METTL1 treated group”, “Cis+OE-M+OE-S” represented “Cisplatin plus overexpressed METTL1 and overexpressed S100A4 treated group”). “*” means *p* < 0.05, “**” means *p* < 0.01 and “NS” means no statistical significance.

The colony formation assay results showed that overexpressed METTL1 aggravated the inhibiting effects of high-dose cisplatin treatment on CR-CC cell proliferation, which were abrogated by synergistically upregulating S100A4 (Figure 6A, 6B). Similarly, overexpressed METTL1 decreased the expression levels of Cyclin D1 as well as CDK2, and increased p27 levels in cisplatin treated CR-HCT116 cells (Figure 6C, 6D), CR-SW480 cells (Figure 6E, 6F) and CR-SW620 cells (Figure 6G, 6H), which were also reversed by upregulation of S100A4 (Figure 6C–6H). Consistently, the FCM results showed that the promoting effects of overexpressed METTL1 on the apoptosis ratio of cisplatin treated CR-CC cells were also reversed by upregulation of S100A4 (Figure 7A, 7B), which were further validated by the Western Blot results by determining the expression levels of cleaved Caspase 3 (Figure 7C, 7D).

## DISCUSSION

Chemoresistance of CC cells to cisplatin seriously limited the utilization and therapeutic efficiency of this chemotherapeutic drug for CC treatment in clinic [3–5], and uncovering the underlying mechanisms might help to solve this problem. Methyltransferase-like 1 (METTL1) was the best characterized enzyme mediating m7G methylation [6–8], which was crucial for the regulation of cancer development [9, 10] and chemosensitivity [9]. In addition, microRNAs could be regulated by METTL1 in a m7G dependent manner [22]. MiR-149-3p served as a tumor suppressor in multiple cancers [14–17], and upregulated METTL1 increased miR-149-3p levels in lung cancer cells in a m7G dependent manner [10]. However, it is still unclear whether METTL1 and miR-149-3p participated in the regulation of CC cells’ chemoresistance to cisplatin. This study found that both METTL1 and miR-149-3p were low-expressed in CR-CC cells comparing to the CS-CC cells. Besides, overexpressed METTL1 increased miR-149-3p levels in CR-CC cells, but upregulation of miR-149-3p did not affect METTL1. levels, which indicated that miR-149-3p was the downstream target of METTL1 and in accordance with the previous study [10]. However, further experiments are still needed to explore whether METTL1 regulated miR-149-3p levels in colon cancer in a m7G dependent manner.

Further results showed that overexpressed METTL1 enhanced the cytotoxic effects of high-dose cisplatin on CR-CC cells, which indicated that upregulation of METTL1 enhanced the chemosensitivity of CR-CC cells to cisplatin. The above results were in accordance with the previous studies [9, 10] and indicated that METTL1 sensitized CR-CC cells to cisplatin treatment. Besides, miR-149-3p was the downstream target of METTL1 [10], and overexpression of miR-149-3p decreased chemoresistance of multiple cancers to cisplatin, such as ovarian cancer [18], esophageal cancer [19], gastric cancer [20] and non-small cell lung cancer [21]. Of note, this study found that the cytotoxic effects of overexpressed METTL1 on CR-CC cells treated with high-dose cisplatin were all abrogated by synergistically knocking down miR-149-3p, which suggested that overexpressed METTL1 sensitized CR-CC cells to high-dose cisplatin treatment by upregulating miR-149-3p and in line with the previous studies [10, 18–21].

S100A4 participated in the regulation of cisplatin-resistance of different cancer cells in a p53-dependent manner [27, 28], and upregulation of S100A4 promoted p53 protein degradation [28]. This study showed that overexpressed S100A4 and downregulated p53 were observed in CR-CC cells compared to CS-CC cells. In addition, overexpressed S100A4 mediated p53 degradation in CR-CC cells, which was in line with the previous study [28]. Notably, either downregulated S100A4 or upregulated p53 promoted high-dose cisplatin induced CR-CC cells death, which suggested that targeting S100A4/p53 axis increased chemosensitivity of CR-CC cells to cisplatin treatment and validated by the previous studies [27, 28]. Furthermore, S100A4/p53 axis was the downstream target of miR-149-3p [15], and overexpressed METTL1 inactivated S100A4/p53 axis in a miR-149-3p dependent manner. Besides, upregulation of S100A4 abrogated the cytotoxic effects of overexpressed METTL1 on CR-CC cells treated with high-dose cisplatin, which suggested that upregulation of METTL1 increased chemosensitivity of CR-CC cells to cisplatin by downregulating S100A4.

Taken together, this study found that overexpressed METTL1 increased chemosensitivity of CR-CC cells to cisplatin by regulating miR-149-3p/S100A4/p53 signaling pathway. Our work will provide new therapeutic strategies for CC treatment in clinic.

## MATERIALS AND METHODS

### Cell culture and induction of Cisplatin-resistant CC (CR-CC) cells

Colon cancer (CC) cell lines (HCT116, SW480 and SW620) and HEK-293T cells were purchased from American Type Culture Collection (ATCC, USA). All the cells were cultured in the Dulbecco’s modified Eagle medium (DMEM, Gibco Company, USA) containing 10% fetal bovine serum (FBS, Gibco Company, USA). The cells were cultured under the standard conditions with humidified atmosphere containing 5% CO_2_ at 37°C. The CC cell lines were continuously exposed to low-dose cisplatin starting from 0.5μg/ml to 5μg/ml to generate cisplatin-resistant CC (CR-CC) cells according to the previous study [1]. After that, the above cells were collected and exposed to high-dose cisplatin (20μg/ml) to verify successful induction of CR-CC cells. The cisplatin-resistant CC (CR-CC) cells were named as CR-HCT116, CR-SW480 and CR-SW620 respectively. Similarly, the cisplatin-sensitive CC (CS-CC) cells were named as CS-HCT116, CS-SW480 and CS-SW620.

### Vectors transfection

The cDNA fragments of METTL1, S100A4 and p53 were prepared by using the EcoRV/Xhol double enzymes digestion method. The above cDNAs sequences were next cloned into the pcDNA3.1 vectors to generate overexpressed METTL1 (OE-METTL1), S100A4 (OE-S100A4) and p53 (OE-p53) vectors respectively. The small interfering RNAs (siRNAs) for S100A4 were obtained from Sangon Biotech (Shanghai, China). In addition, the miR-149-3p mimic and inhibitor were also synthesized by Sangon Biotech (Shanghai, China). The above vectors were transfected into CR-CC cells by using the Lipofectamine 2000 reagent (Invitrogen, CA, USA) according to the manufacturer’s protocol. After 24 hours post-transfection, Real-Time qPCR was used to detect the transfection efficiency.

### Real-time qPCR

The CR-CC cells (CR-HCT116, CR-SW480 and CR-SW620) and CS-CC cells (CS-HCT116, CS-SW480 and CS-SW620) were collected and the total RNA were extracted by using the TakaRa MiniBEST Universal RNA Extraction Kit (Takara, Japan) according to the manufacturer’s protocol. The total RNA were dissolved in the diethyl pyrocarbonate (DEPC) water and reversely transcribed into complementary DNA (cDNA) by using the TaqMan Reverse Transcription Reagents (Applied Biosystems, USA). After that, the SYBR Green SuperMix Kit (Takara, Japan) was used to amplify and quantify the involved genes including miR-149-3p and the mRNA levels of METTL1, S100A4 as well as p53. The primer sequences for the above genes were listed in Table 1.

**Table 1:** Primer sequences for Real-Time qPCR.

**Gene**	**Primer sequences (strand)**
β-actin	Forward: 5′-CTCCATCCTGGCCTCGCTGT-3′ Reverse: 5′-GCTGCTACCTTCACCGTTCC-3′
U6	Forward: 5′-GACTATCATATGCTTACCGT-3′ Reverse: 5′-GGGCAGGAAGAGGGCCTAT-3′
miR-149-3p	Forward: 5′-GGCTCTGGCTCCGTGTCTT-3′ Reverse: 5′-CAGTGCAGGGTCCGAGGTATT-3′
METTL1	Forward: 5′-GAACATCGCCTGTCTCCGAA-3′ Reverse: 5′-TCGCTTAAAGTGTGGGTCCG-3′
S100A4	Forward: 5′-GATGTGATGGTGTCCACCTT-3′ Reverse: 5′-ATTTCTTCCTGGGCTGCTTA-3′
p53	Forward: 5′-CAGCCAAGTCTGTGACTTGCACGTAC-3′ Reverse: 5′-CTATGTCGAAAAGTGTTTCTGTCATC-3′

The genes included β-actin, U6, miR-149-3p, METTL1, S100A4 and p53.

### Western blot

The CR-CC and CS-CC cells were collected and the total proteins were extracted by using the RIPA lysis buffer purchased from Beyotime Biotechnology (Shanghai, China). The 10% sodium dodecyl sulfate (SDS)-polyacrylamide gel electrophoresis (PAGE) was next employed to separate the target proteins according to their molecular weight. After that, the separated proteins were transferred to the poly vinylidene difluoride (PVDF) membranes and blocked by 5% skim milk solution. The membranes were probed with the primary antibodies including anti-METTL1 (1:1000, Abcam, UK), anti-β-actin (1:2000, Abcam, UK), anti-Cyclin D1 (1:1500, Abcam, UK), anti-CDK2(1:1500, Abcam, UK), anti-p27 (1:1000, Abcam, UK), anti-cleaved Caspase 3 (1:1000, Abcam, UK), anti-S100A4 (1:1000, Abcam, UK) and anti-p53 (1:1000, Abcam, UK) for 2 hours at room temperature. The secondary antibody purchased from Abcam (1:1000) was sequentially incubated with the PVDF membranes. The protein bands were visualized by using the ECL luminescence reagent (ThermoFisher Scientific, USA) and quantified by performing the Image J software (NIH, USA).

### Cell counting kit-8 (CCK-8) assay

The CR-CC cells (CR-HCT116, CR-SW480 and CR-SW620) and CS-CC cells (CS-HCT116, CS-SW480 and CS-SW620) were all cultured under the standard cuture conditions. The above vectors were transfected into the cells until the cell confluency reached about 60-70%. The transfection efficiency of the vectors were determined by using the Real-Time qPCR and Western Blot technologies. About 24 hours post-transfection, the cells were collected and seeded into the 96-well plates at the density of 3000 cells per well. After 4 hours, the cells were incubated with high-dose cisplatin (20μg/ml) for 0h, 24h, 48h, 72h and 96h respectively. The CCK-8 kit (YEASEN, Shanghai, China) was used to determine cell proliferation according to the manufacturer’s instruction. Briefly, 10μl of the CCK-8 solution were added to each well and incubated with the cells for 2 hours. After that, the optical density (OD) values in the wavelength of 450nm were detected to evaluate cell proliferation.

### Colony formation assay

The CR-CC cells (CR-HCT116, CR-SW480 and CR-SW629) were transfected with the above vectors for 24 hours and collected for further experiments. The cells were plated in 24-well plates at the density of 1000 cells per well, and cultured routinely at the standard conditions for 14 days according to the previous study [32]. After that, the cells were fixed with paraformaldehyde and stained with crystal violet (Beyotime Technology, Shanghai, China) for 10 min. Finally, the colonies containing at least 10 cells were counted under the light microscopy.

### Flow cytometry (FCM)

The CR-CC cells (CR-HCT116, CR-SW480 and CR-SW629) were treated with high-dose cisplatin for 48 hours and cells were collected for apoptosis analysis. The Annexin V/Propidium iodide (PI) double stain kit (HaiGene Corporation, China) were purchased to determine the apoptosis ratio of the above cells according to the manufacturer’s instruction. In brief, the above cells were diluted into cell suspensions at the density of 1×10^4^/ml. The cell suspensions were next incubated with the Annexin V-FITC and PI double staining solution for 15-20 min at room temperature without light. Finally, the FCM machine (BD Biosciences, NJ, USA) was employed to determine the apoptosis ratio of the above cells.

### Dual-luciferase reporter gene system

The online StarBase software (http://starbase.sysu.edu.cn/) was used to predict the binding sites of miR-149-3p and 3′UTR regions of S100A4. The 3′UTR regions of S100A4 were mutated according to the S100A4 sequences. After that, the wild-type (Wt) and mutant (Mut) 3′UTR regions of S100A4 were synthesized and cloned into the pmirGLO Expression Vector by Sangon Biotech (Shanghai, China) to generate Wt-S100A4 and Mut-S100A4 vectors respectively. Besides, the miR-149-3p mimic and inhibitor were obtained from Sangon Biotech (Shanghai, China). The above vectors were co-transfected into the HEK-293T cells by using the Lipofectamine 2000 reagent (Invitrogen, CA, USA) according to the manufacturer’s protocol. After 24 hours post transfection, the luciferase activities were detected by using the dual-luciferase reporter gene system (Promega, USA) to validate the binding sites of miR-149-3p and 3’UTR regions of S100A4.

### Terminal Deoxynucleotidyl Transferase (TdT)-mediated dUTP Nick-End Labeling (TUNEL) assay

The TUNEL assay was performed to determine cell apoptosis by using a TUNEL fluorescence kit (Rohce, Switzerland) according to the manufacturer’s protocol. Briefly, the CR-CC cells were fixed with 4% paraformaldehyde for 30 min at room temperature. The cell membranes were next broken by treating cells with phosphate buffer saline (PBS) buffer containing 0.3% Triton X-100 (PBST), and the TdT mixed with dUTP was added to the cells. Finally, the cells were stained with DAPI for nuclei, and the Laser Scanning Confocal Microscope (Leica, Japan) was employed to determine cell apoptosis ratio according to the percentages of TUNEL positive cells.

### Statistical analysis

All the data were collected and represented as Mean ± Standard Deviation (SD). The data analysis was conducted by using the SPSS 18.0 software. Specifically, the student’s t-test was used to compare the two groups, and the ANOVA was employed to analyse the differences among multiple groups. “*P* < 0.05” was regarded as statistical significance.

## Supplementary Material

Supplementary Figures
