# Supplementary material for: Overexpressed methyltransferase-like 1 (METTL1) increased chemosensitivity of colon cancer cells to cisplatin by regulating miR-149-3p/S100A4/p53 axis
Source: Aging (Albany NY). 2019 Dec 20;11(24):12328–44. doi: 10.18632/aging.102575 (PMC6949057; doi:10.18632/aging.102575)
Supplement: Supplementary Figures [file aging-11-102575-s001..pdf]

SUPPLEMENTARY FIGURES

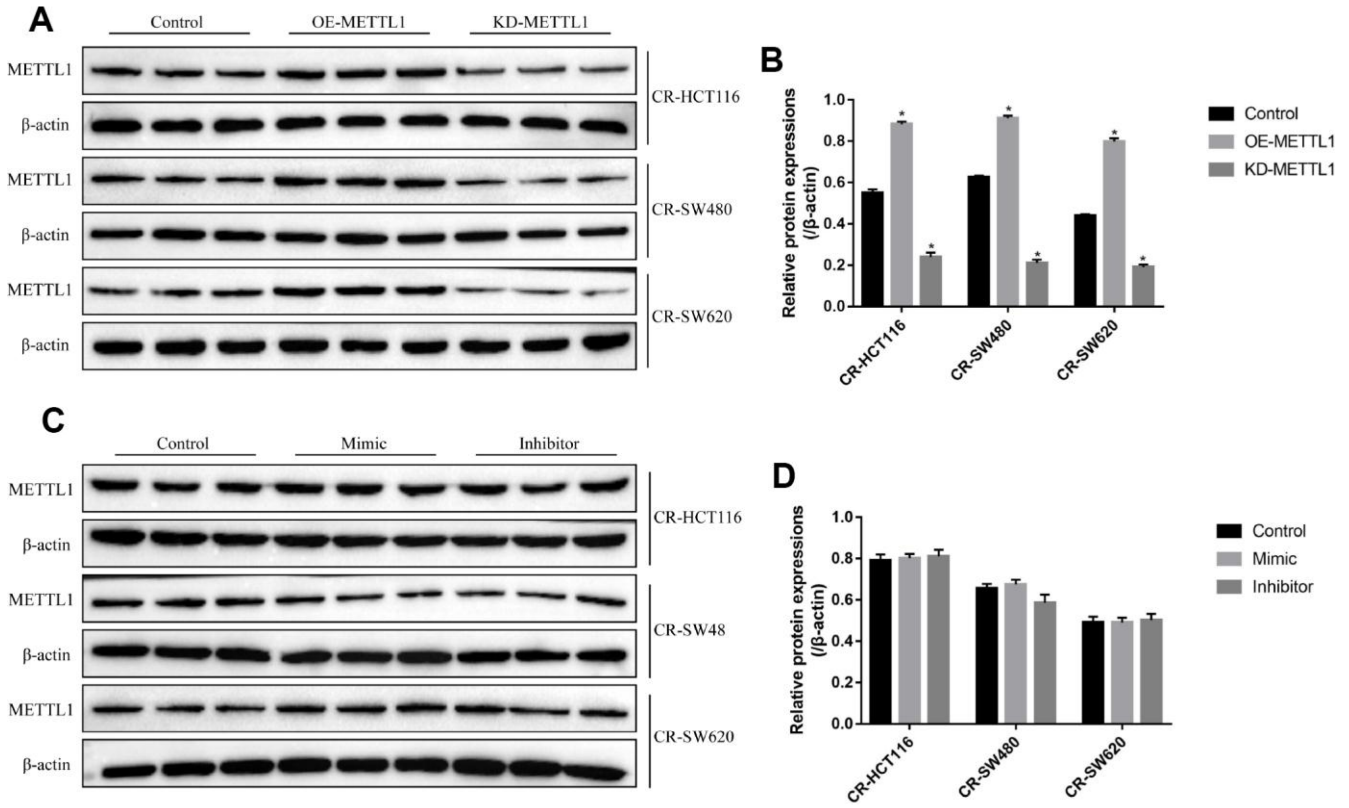

Supplementary Figure 1. Western Blot was used to determine (**A**) the expression levels of METTL1 in CR-CC cells and (**B**) the effects of miR-149-3p on METTL1 levels in CR-CC cells. All the experiments repeated at least 3 times. "*" means $p < 0.05$, "**" means $p < 0.01$ and "NS" means no statistical significance.

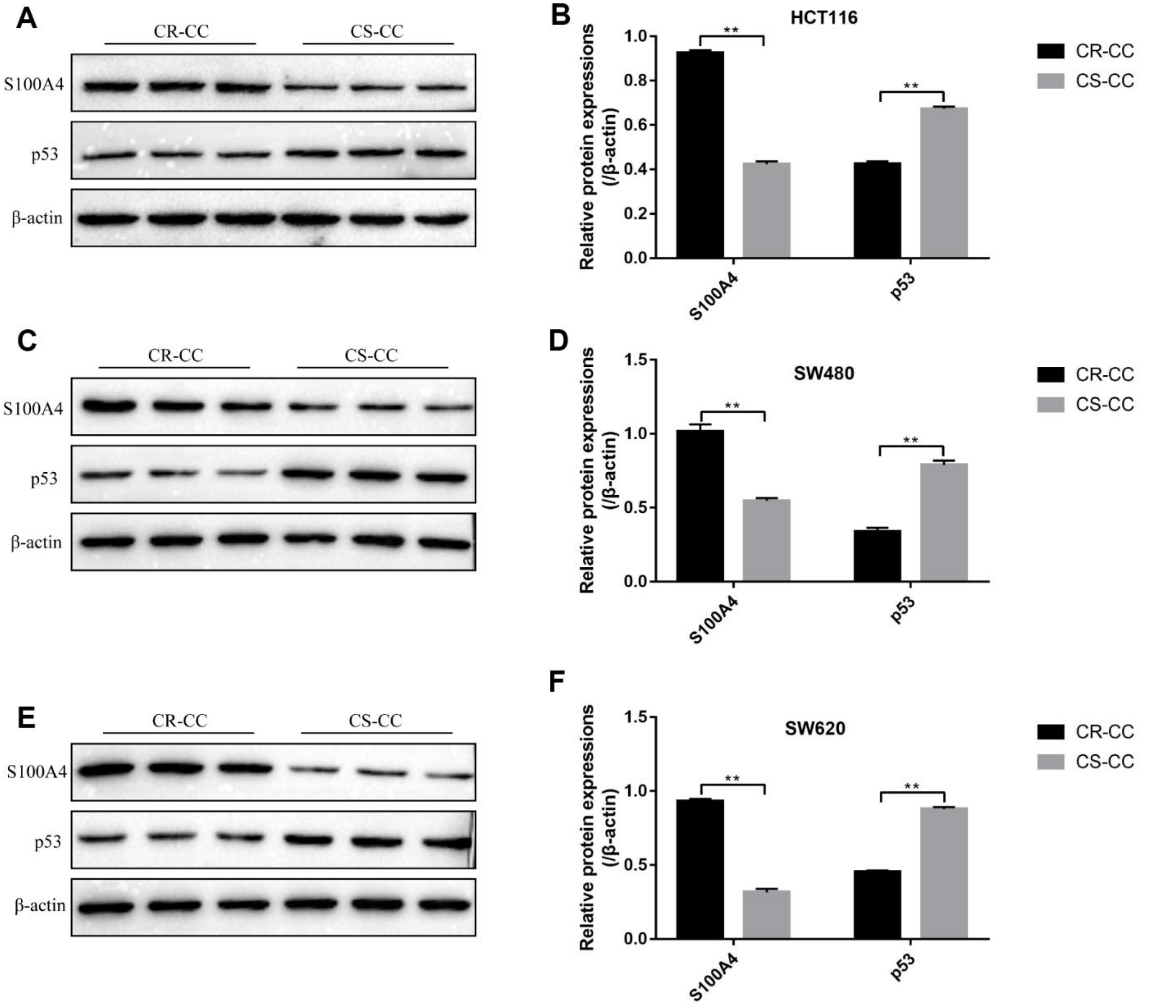

Supplementary Figure 2. Western Blot was used to determine the expression levels of S100A4 and p53 in (**A**, **B**) HCT116 cells, (**C**, **D**) SW480 cells and (**E**, **F**) SW620 cells. All the experiments repeated at least 3 times. "*" means $p < 0.05$, "**" means $p < 0.01$ and "NS" means no statistical significance.

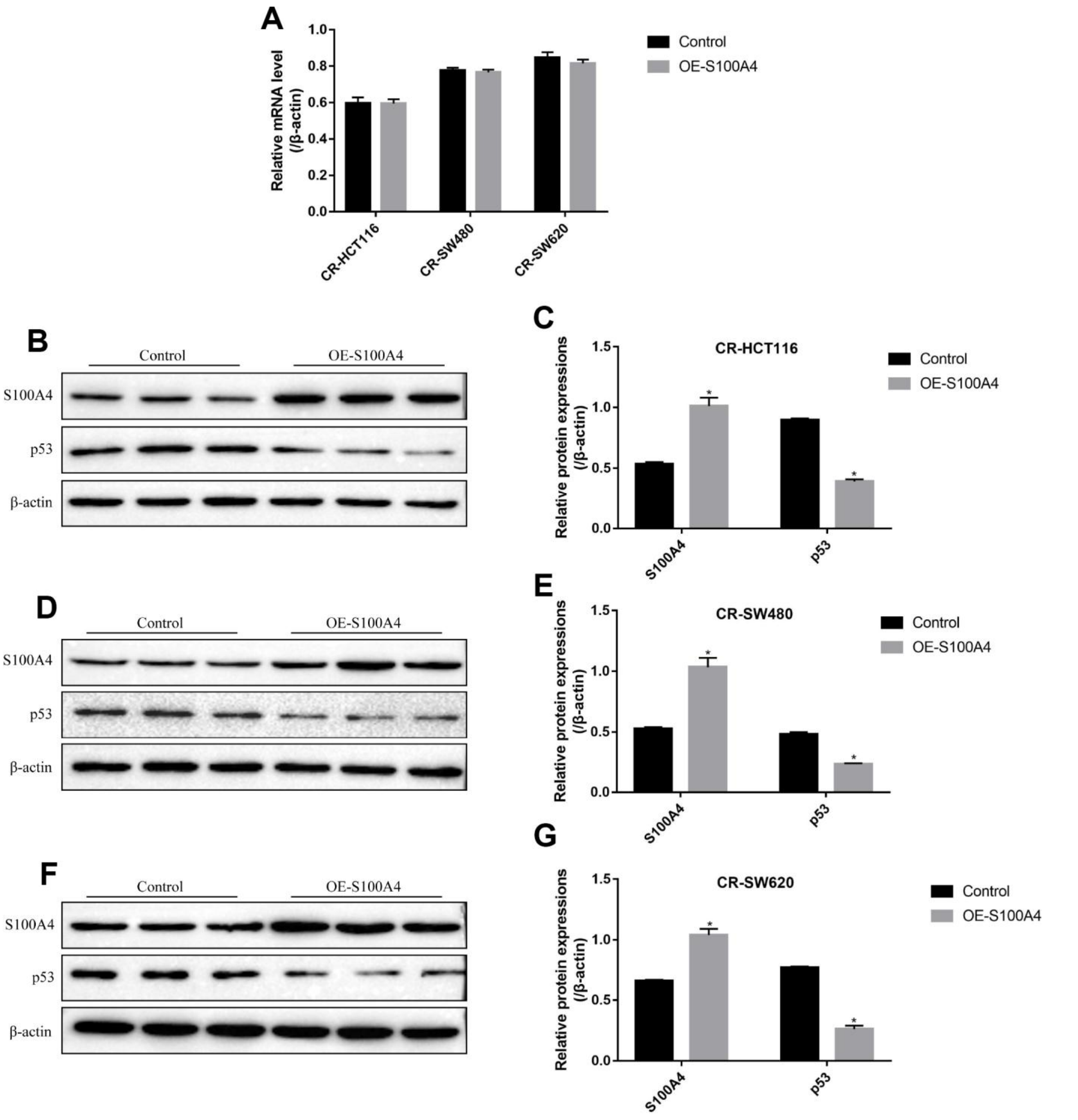

Supplementary Figure 3. The effects of S100A4 on p53 levels in CR-CC cells. (**A**) Real-Time qPCR was used to detect S100A4 mRNA levels. Western Blot was used to determine the expression levels of S100A4 and p53 in (**B**, **C**) CR-HCT116 cells, (**D**, **E**) CR-SW480 cells and (**F**, **G**) CR-SW620 cells. All the experiments repeated at least 3 times. “*” means $p < 0.05$, “**” means $p < 0.01$ and “NS” means no statistical significance.
